# Effect of Otago exercise on fear of falling in older adults: a systematic review and meta-analysis

**DOI:** 10.1186/s13102-024-00917-2

**Published:** 2024-06-14

**Authors:** Jianlong Han, Hongwei Wang, Yunxin Ding, Qing Li, Huanqie Zhai, Shuling He

**Affiliations:** 1 Jiamusi University, Jiamusi, China; 2 The Fourth Affiliated Hospital of Harbin Medical University, Harbin, China; 3 The First Affiliated Hospital of Jiamusi University, Jiamusi, China

**Keywords:** Otago exercise programme, Older adults, Fear of falling, Meta-analysis

## Abstract

**Background:**

Approximately 40–70% of older adults who have experienced falls develop fear of falling (FOF), with the incidence rate in nursing home residents reaching as high as 79.4%. An increasing number of studies have focused on the effect of the Otago Exercise Programme (OEP) on reducing FOF among older adults, yet comprehensive analysis is lacking due to regional and demographic variations. Therefore, this study integrates the relevant literature to provide evidence supporting interventions aimed at alleviating FOF among older adults.

**Objective:**

To evaluate the impact of OEP on FOF in older adults through meta-analysis.

**Methods:**

We searched ten databases using computer systems, covering all records up to May 1, 2024. Two researchers independently conducted the literature screening, bias risk assessment, and data extraction. We performed data analysis using RevMan 5.3 and Stata 15.0 software, assessed result stability through sensitivity analysis, and examined publication bias with funnel plots and Egger’s test.

**Results:**

Sixteen RCTs were included. Meta-analysis revealed that the OEP significantly reduced FOF among older adults [SMD = 0.96, 95%CI (0.68, 1.23), *P* < 0.00001]. Subgroup analysis revealed that interventions lasting more than 16 weeks [SMD = 1.12, 95%CI (0.75, 1.49), *P* < 0.00001], with a frequency of more than twice a week [SMD = 0.99, 95%CI (0.64, 1.35), *P* < 0.00001], and for older adults in community and nursing institutions [SMD = 1.03, 95%CI (0.50, 1.57), *P* = 0.0002] were more effective. A comparison of the 16-week and 24-week interventions revealed that the latter had better outcomes [SMD = 0.87, 95%CI (0.66, 1.08), *P* = 0.0004].

**Conclusion:**

Current evidence indicates that OEP effectively reduces FOF among older adults. It is recommended that interventions last for more than 24 weeks, occur more than twice a week, and suitable for application among older adults in community settings or elder care institutions.

## Introduction

Fear of Falling (FOF), refers to the avoidance of daily activities due to the fear of falling, such as cleaning, which can lead to symptoms such as anxiety and palpitations if these activities are undertaken [1]. FOF is prevalent among older adults, Xiong et al. reported that fear of falling occurs in approximately 49.6% of the population worldwide, with a maximum prevalence of 90.34%, and that the prevalence is high in developing countries and disease groups [2]. The relationship between FOF and actual falls is closely linked, with each influencing the other in a vicious cycle [3]. Ho LYW et al. conducted a cross-sectional survey on older stroke patients, and their results showed that severe fear of falling can increase the incidence of falls, restrict daily living activities, and lead to frailty [4]. Excessive worry about the consequences of falling can reduce social interactions, and trigger negative emotions such as anxiety and depression, severely affecting quality of life [5]. In a global initiative proposed by Montero-Odasso et al. in 2022 on the prevention and management of falls in older adults, it is recommended to reduce the fear of falling in older adults through exercise [6]. The Otago Exercise Programme (OEP) was developed by Campbell and others [7]. OEP through warm-up exercises, strength training, balance training, and walking activities, can be used to develop personalized training plans based on the physical condition of older adults [8]. Gradual and systematic exercise increases the sensitivity of proprioceptors in older adults thereby improving physical function and reducing FOF [9]. Currently, relevant meta-analyses have explored the effects of OEP on older adults [10–12]. Their results indicate that OEP can enhance lower limb strength and balance in older adults. However, there is currently a lack of attention to the psychological issues of falls in older adults, and no studies have deeply explored the impact of OEP on the FOF in older adults. Yu et al.‘s findings suggest that OEP can reduce FOF in older adults [11], but their work involved only a descriptive analysis of two studies, lacking in-depth investigation. Therefore, to explore in depth the effects of OEP on FOF in older adults, this study integrates and analyzes related literature through meta-analysis to explore the effects of different intervention durations and frequencies, and to provide evidence supporting the application of OEP in the older adult population.

## Methods

### Research design

This study is based on the PICOS framework. This study followed the Preferred Reporting Items for Systematic Reviews and Meta-Analyses (PRISMA2020) guidelines for systematic reviews and meta-analyses. The study was retrospectively registered on the PROSPERO platform (CRD42024529549).

### Literature search

We conducted searches in four commonly used Chinese databases: CNKI, Wanfang, VIP, and the China Biology Medicine disc (CBM), as well as five English databases: CINAHL, Embase, Cochrane Library, Web of Science, and PubMed. We retrieved all studies available up to May 1, 2024, using the search terms “otago exercise” “otago” " OEP” " Aged” " Aging” “elderly” “old people” “seniors” “fear of falling” “FOF” and “fear”tailored to the requirements of each database. This was supplemented by manual searches to screen the references of the included literature for selection.

### Inclusion and exclusion criteria

Based on the PICOS principle, we established the inclusion criteria. (1) The inclusion criteria were as follows; (i) study subjects: older adults aged 60 years and older; (ii) intervention measures: the control group received conventional exercise training or care, while the intervention group received OEP in addition to the control group’s regimen; (iii) outcome indicators: FOF; (iv) study type: randomized controlled trials (RCTs). The exclusion criteria include: (i) studies combining other interventions; (ii) studies that were duplicate publications or for which the full text was not available.

### Screening and data extraction

We imported all literature into EndNote X9 software for screening, which was performed independently by two researchers. The authors excluded duplicates, studies with nonmatching intervention measures, and nonmatching intervention subjects by reading the study titles. By reading the literature abstracts and full texts, they excluded non-RCT literature, studies with nonmatching outcome indicators, and literature for which the full text was not accessible. After completing their selections, the two researchers cross-checked their results. Any disagreements were resolved by a third researcher. Two researchers then extracted the data according to a predesigned data extraction form. The extracted content included the authors’ names, publication year, intervention population, sample size, intervention measures, control measures, duration of a single intervention, intervention period and frequency, and the evaluation tools and results for the outcome indicators. Upon completion, the two researchers cross-checked their work. Any disagreements were discussed and resolved with the involvement of a third researcher.

### Risk of literature bias and assessment of publication bias

Two researchers independently assessed the risk of bias in the included studies using the risk of bias assessment tool for randomized controlled trials recommended by the Cochrane Collaboration [13]. They evaluated seven aspects, each of which were rated as low risk (+), unclear risk (?), or high risk (-). Studies that fully met the criteria were classified as Grade A, those that partially met the criteria as Grade B, and those that did not meet the criteria at all as Grade C. We used funnel plots and Egger’s test to assess whether there was any publication bias in this study. A t value in Egger’s test was greater than 0.05, according to Egger’s test indicated no significant publication bias. We used the GRADE method to rate the quality of evidence for the results [13], with evidence levels being high, medium, low, and very low.

### Statistical methods

We conducted a meta-analysis using Review Manager 5.3 and Stata 17.0 software. Given the wide range of sources of heterogeneity and their impact on the results, we used the standardized mean difference (SMD) and 95% confidence intervals (CI) as the effect sizes for this study. SMD values of 0.2, 0.5, and 0.8 indicate minor, moderate, and large significant effects, respectively [12]. We assessed the magnitude of heterogeneity using the I² statistic and P-value. For studies with low heterogeneity (I²≤50%, *P* > 0.1), we performed a meta-analysis using a fixed-effects model. For studies with high heterogeneity (I²≥50%, *P* < 0.1), we employed a random-effects model. In cases of high heterogeneity, we conducted subgroup analyses to identify sources of heterogeneity and to determine the most suitable frequency and duration of exercise for older adults. We considered P-values < 0.05 to indicate statistical singnificance.

### Sensitivity analysis

We performed sensitivity analysis with alternating effect models and sequentially excluded the included studies. If the effect size significantly changes upon switching models or removing a specific study, the results of our study may be unstable.

## Results

### Literature screening results

**Fig. 1:**
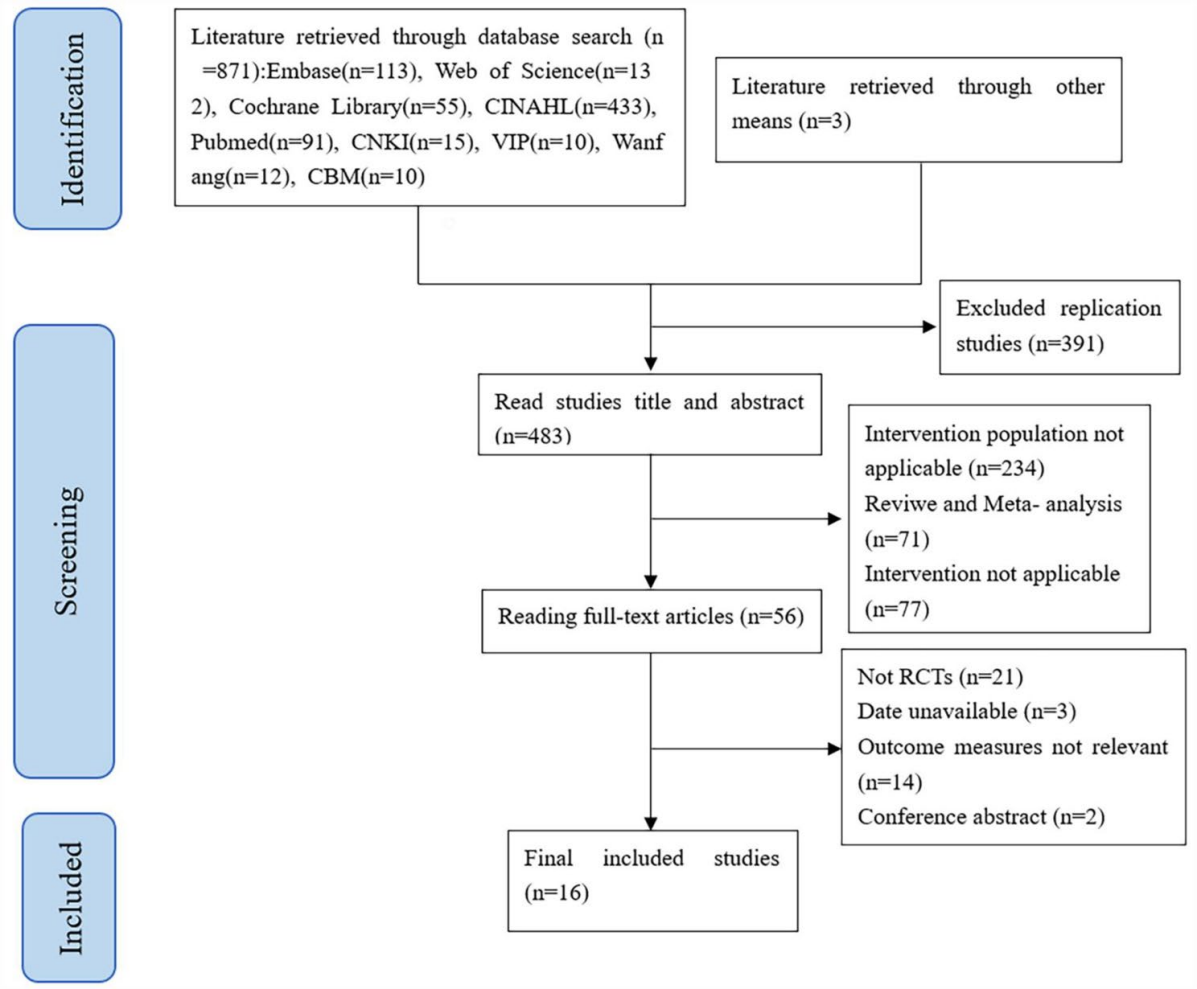
Literature screening flowchart

A total of 871 studies were identified. Finally, 16 studies were included [14–29]. The screening process is shown in Fig. 1.

### Literature characteristics and bias risk assessment

**Fig. 2:**
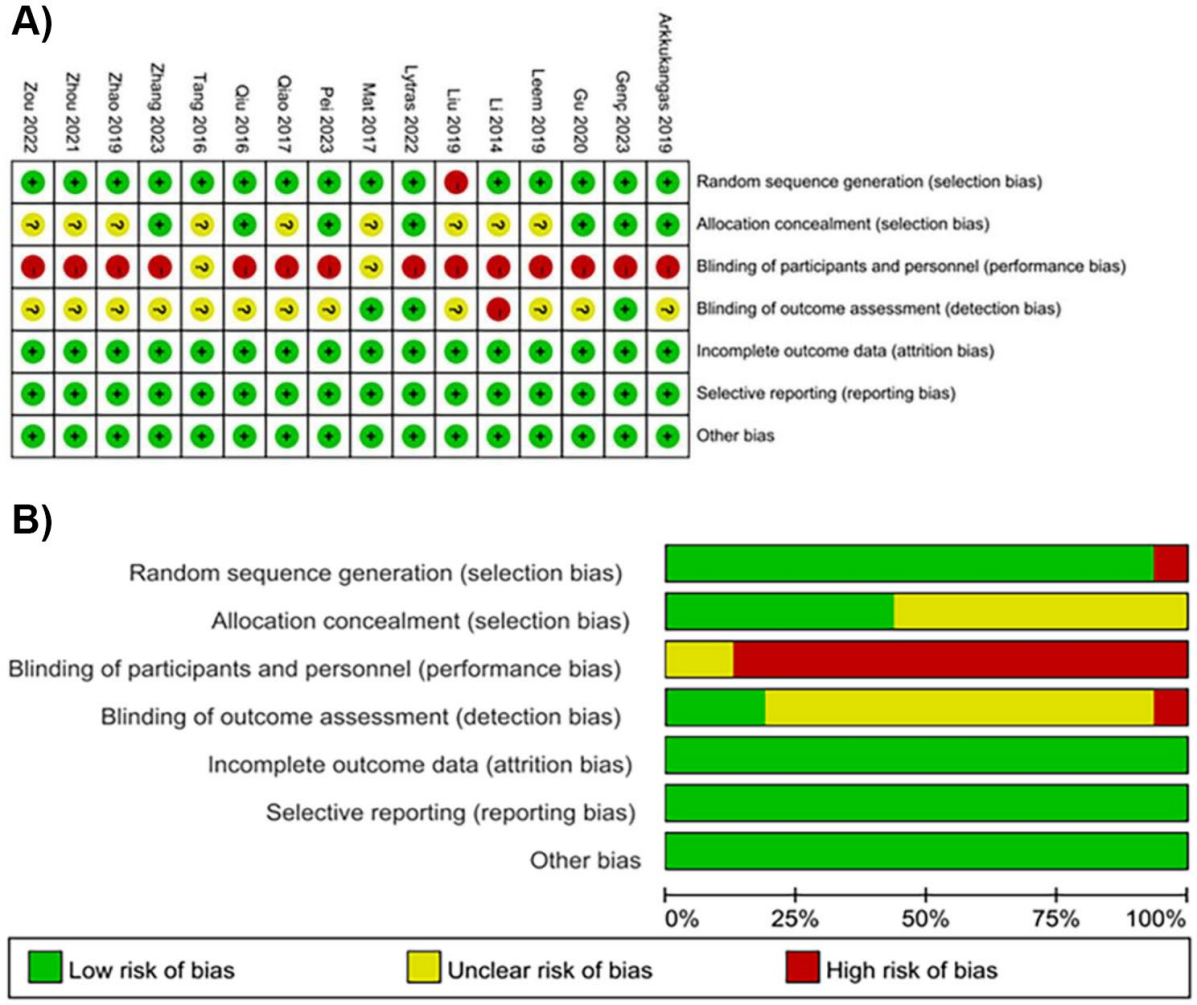
**a** Risk of bias assessment chart. **b** Risk of bias assessment chart (expressed as percentages)

Studies from China [19–29], Turkey [15], Greece [17], Malaysia [18], Korea [16], and Sweden [14], involved a total of 1290 older adults, 644 of whom were receiving OEP training. The study population mainly consisted of community-dwelling older adults and older stroke patients. Details on the intervention content, frequency, and duration are available in Table 1. All sixteen studies received a Grade B rating. One study described random allocation without detailing the method of randomization [16], leading us to assess it as high risk, while seven studies concealed the method of random allocation [13–16, 20, 22, 24, 26], assessed it as low risk. Three studies described the blinding of assessors [15, 20, 21], which we evaluated as low risk. The results of the literature bias risk assessment are presented in Fig. 2a and b.

**Table 1:** Characteristics of included studies

Study	Country	Population source	Participants	Intervention group	Control group	Intervention duration, cycle and frequency	Outcome measures assessment tools
Arkkukangas 2019 [14]	Sweden	Community elderly	T:54 C:55	OEP(Training plan with gradual intensification based on individual situation)	Routine drill	12 weeks, 3 times a week, 30 minutes each time	①
Genç 2023 [15]	Turkey	Elderly people in nursing institutions	T:75 C:75	OEP exercises (warm-up, strengthening, balance, walking program)	Routine drill	12 weeks, 3 times a week, 30 minutes each time	②
Leem 2019 [16]	Korea	Social recruitment of the elderly	T:10 C:10	OEP exercises (Strength training, balance training)	Routine drill	12 weeks, 3 times a week, 40 minutes each time	③
Lytras 2022 [17]	Greece	Elderly people in nursing institutions	T:75 C:75	OEP training (warm-up exercises, lower limb muscle resistance exercises, improving dynamic and static balance exercises, range of motion exercises, recovery exercises)	Routine drill	24 weeks, Three times a week for the first three weeks, once a week after that, 45 minutes each time	③
Mat 2017 [18]	Malaysia	Elderly patients with osteoarthritis	T:17 C:17	OEP exercise	Routine drill	24 weeks, 3 times a week, 40 minutes each time	③
Zou 2022 [19]	China	Elderly people in nursing institutions	T:29 C:28	OEP (Otago training phase: warm-up, strength training, balance training. Walking link)	Usual care	12 weeks, 2 times a week, 40–60 minutes each time	④
Qiao 2017 [20]	China	Elderly patients with central hemiplegia	T:30 C:31	OEP training instruction (Otago training: warm-up training, strength training, balance training. Walking exercise)	Routine follow-up	20 weeks, 2–3 times a week, 30 minutes each time	②
Liu 2019 [21]	China	Elderly patients undergoing knee replacement	T:33 C:32	OEP training (warm-up, muscle training, balance training)	Routine drill	4 weeks, 7 times a week, 30 minutes each time	②
Zhou 2021 [22]	China	Hospitalized elderly patients	T:30 C:30	OEP training (warm-up, balance training, muscle training and stretching)	Usual care	4 weeks, 3 times a week, 30 minutes each time	②
Tang 2016 [23]	China	Parkinson’s discharged elderly patients	T:30 C:30	OEP training Guide ( Otago training: warm-up, strength training, balance training)	Routine follow-up	24 weeks, 3 times a week, 30 minutes each time	②
Zhang 2023 [24]	China	Elderly stroke patients	T:35 C:34	OEP training (warm-up runs, strength training, balance training, walking exercises)	Usual care	12 weeks, 2–3 times a week, 30 minutes each time	②
Li 2014 [25]	China	Elderly stroke patients	T:28 C:28	OEP campaign (program development; Home training: warm-up exercise, strength training, balance training, walking exercise; Outpatient follow-up)	Usual care	16 weeks, 3 times a week, 30 minutes each time	②
Pei 2023 [26]	China	Community elderly	T:39 C:40	OEP training (warm-up, strength training, balance training, walking exercises)	Routine follow-up	12 weeks, 3 times a week, 30 minutes each time	②
Zhao 2019 [27]	China	Elderly patients with femoral neck fracture	T:63 C:63	OEP training guidance (Release training videos and guidance; Warm-up exercises, strength training and balance training; Follow-up visit)	Routine follow-up	24 weeks	②
Qiu 2016 [28]	China	Elderly stroke patients	T:66 C:68	OEP training (warm-up, strength training, balance training, walking exercises)	Routine rehabilitation training	16 weeks, 2–3 times a week, 30 minutes each time	②
Gu 2020 [29]	China	Outpatient follow-up of elderly patients	T:30 C:30	OEP training (warm-up runs, strength training, balance training, walking exercises)	Routine follow-up	24 weeks, 3 times a week, 30 minutes each time	⑤

T: treatment group C: control group

①The Falls Effi cacy Scale Swedish version (FES(S)) ②Modified fall efficacy scale ③Falls Efficacy Scale-International (Short FES-I)

④ Modified Survey of Activities and Fear of Falling in the Elderly (mSAFFE) ⑤Autism Behavior Checklist

### Meta-analysis results

**Fig. 3:**
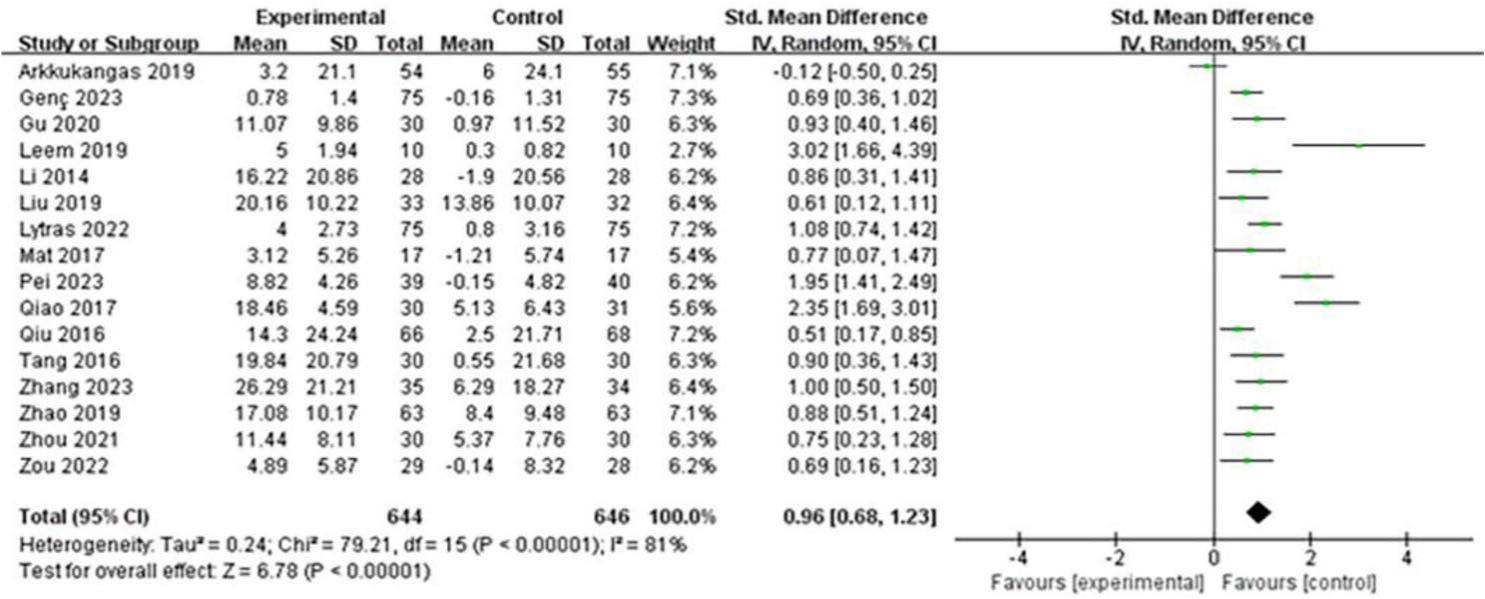
Forest plot of the effect of OEP on fall fear in older adults

The overall meta-analysis results showed heterogeneity among study outcomes (I²=81%, *P* < 0.00001), leading us to use a random-effects model for aggregation. The results indicated [SMD = 0.96, 95%CI (0.68, 1.23), *P* < 0.00001], and the difference was statistically significant (Z = 6.78, *P* < 0.00001), (Fig. 3). The level of evidence was medium.

### Subgroup analysis

**Fig. 4:**
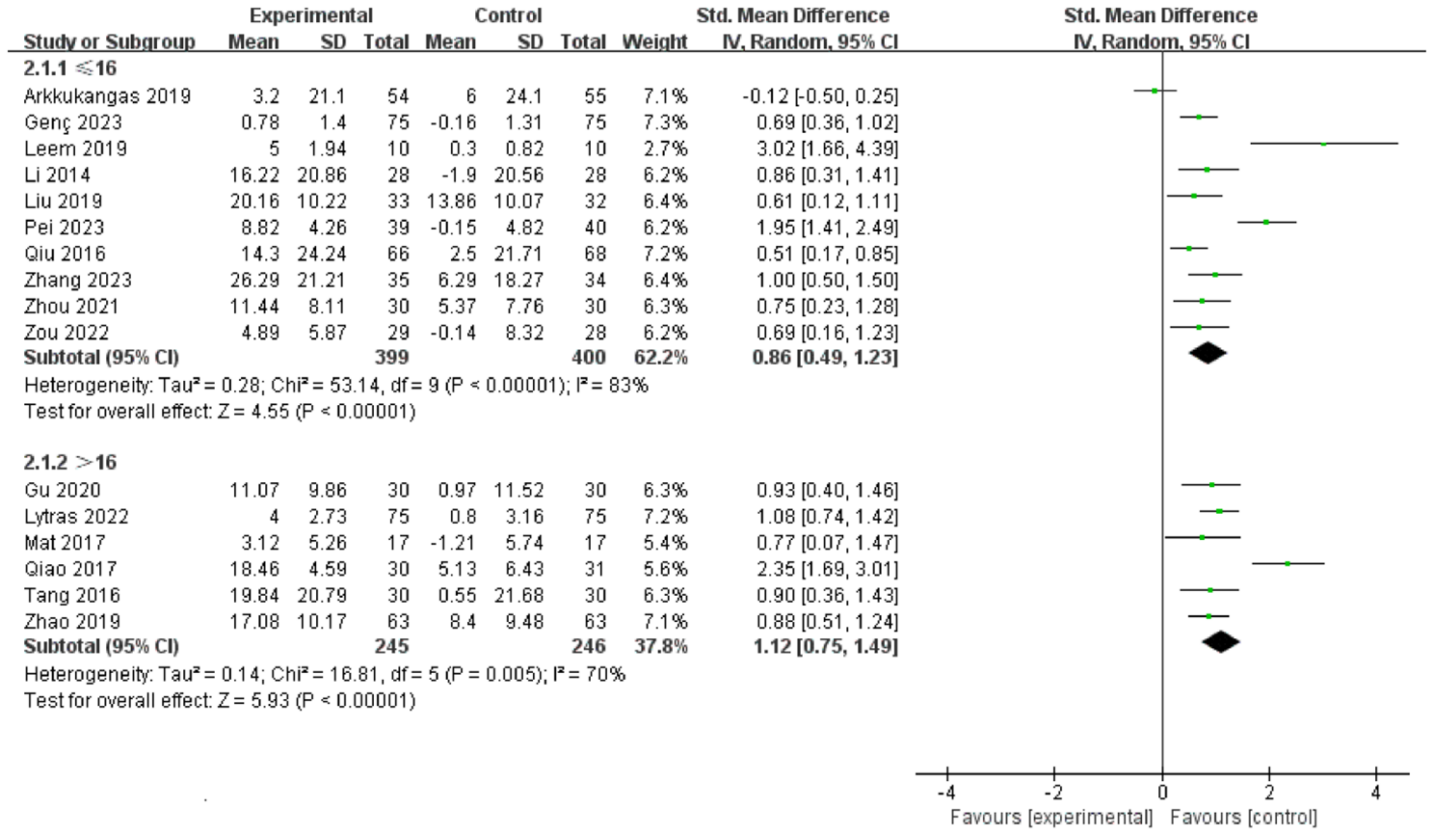
Forest plot of subgroup analysis for ≤ 16 weeks and > 16 weeks

**Fig. 5:**
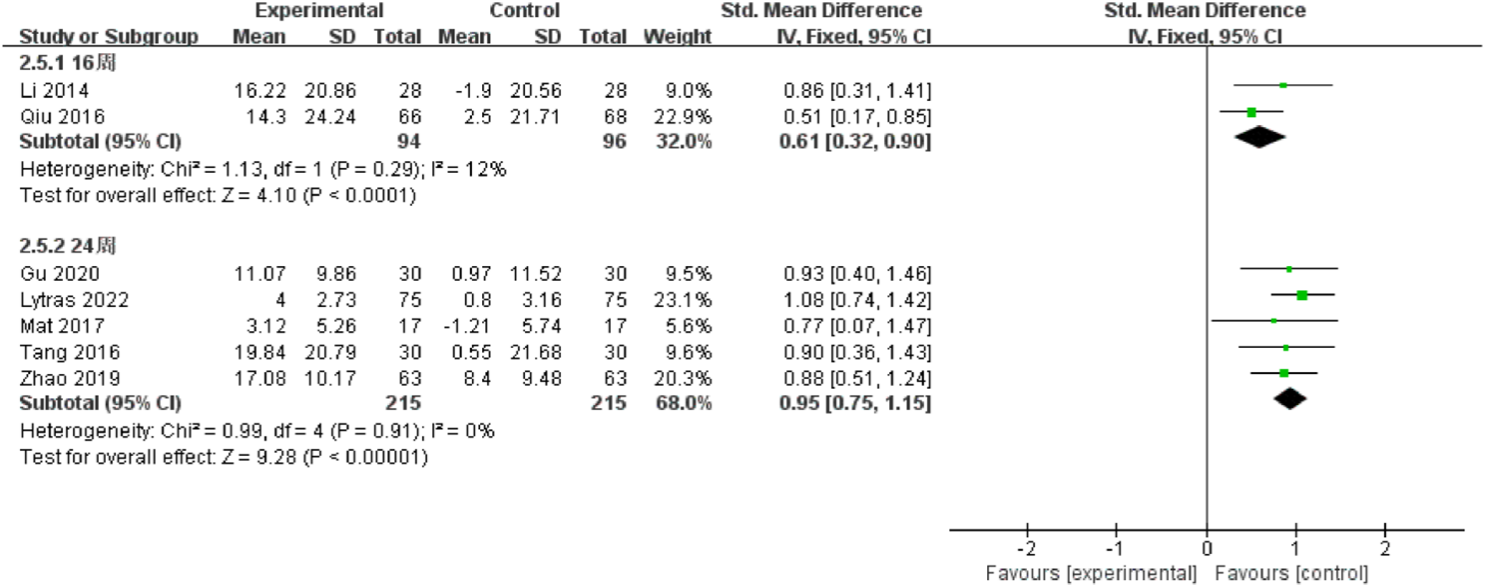
Forest plot of subgroup analysis for 16 weeks and 24 weeks

**Fig. 6:**
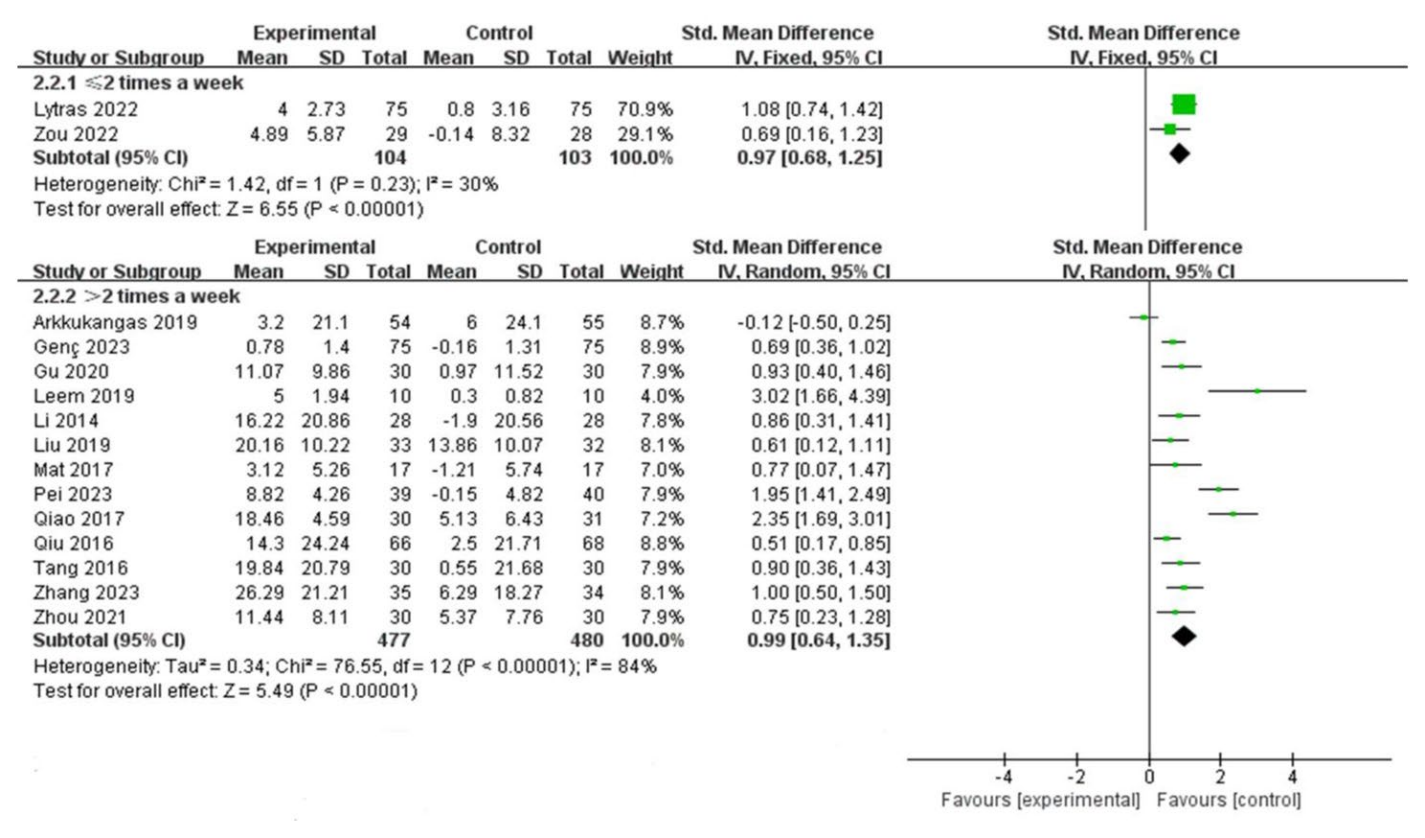
Forest plot of subgroup analysis for intervention frequency

**Fig. 7:**
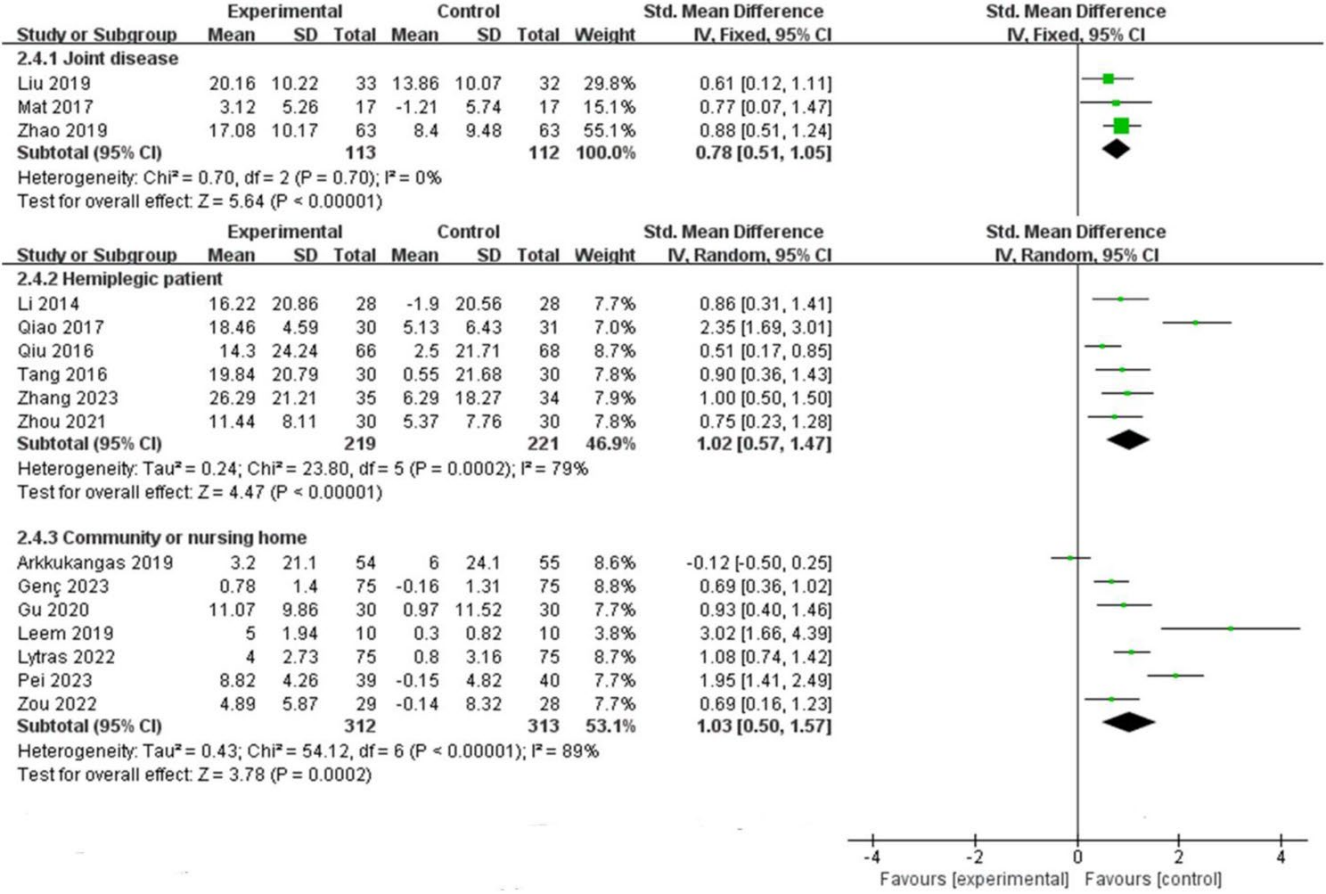
Forest plot of subgroup analysis for intervention populations

Among the 16 included studies, 10 had an intervention duration of ≤ 16 weeks, while 6 had an intervention duration of > 16 weeks. The results indicated that interventions lasting > 16 weeks [SMD = 1.12, 95% CI (0.75, 1.49), *P* < 0.00001] were more effective than those lasting ≤ 16 weeks [SMD = 0.86, 95% CI (0.49, 1.23), *P* < 0.00001], (Fig. 4). Comparing intervention durations of 16 weeks and 24 weeks, the 24-week intervention [SMD = 0.95, 95% CI (0.75, 1.15), *P* < 0.00001] was more effective than the 16-week intervention [SMD = 0.61, 95% CI (0.32, 0.90), *P* < 0.00001], (Fig. 5). Subgroup analysis based on intervention frequency revealed that interventions occurring more than twice a week [SMD = 0.99, 95% CI (0.64, 1.35), *P* < 0.00001] were more effective than those occurring twice or less per week [SMD = 0.97, 95% CI (0.68, 1.25), *P* < 0.00001], (Fig. 6). Subgroup analysis of different populations indicated that the application of OEP was most effective for older adults in communities or nursing homes [SMD = 1.03, 95% CI (0.50, 1.57), *P* < 0.00001], followed by patients with hemiplegia [SMD = 1.02, 95% CI (0.57, 1.47), *P* < 0.00001]. The effectiveness was lowest among older adults with joint diseases [SMD = 0.78, 95% CI (0.51, 1.05), *P* < 0.00001] compared to the previous two groups, (Fig. 7).

### Sensitivity analysis and publication bias

**Fig. 8:**
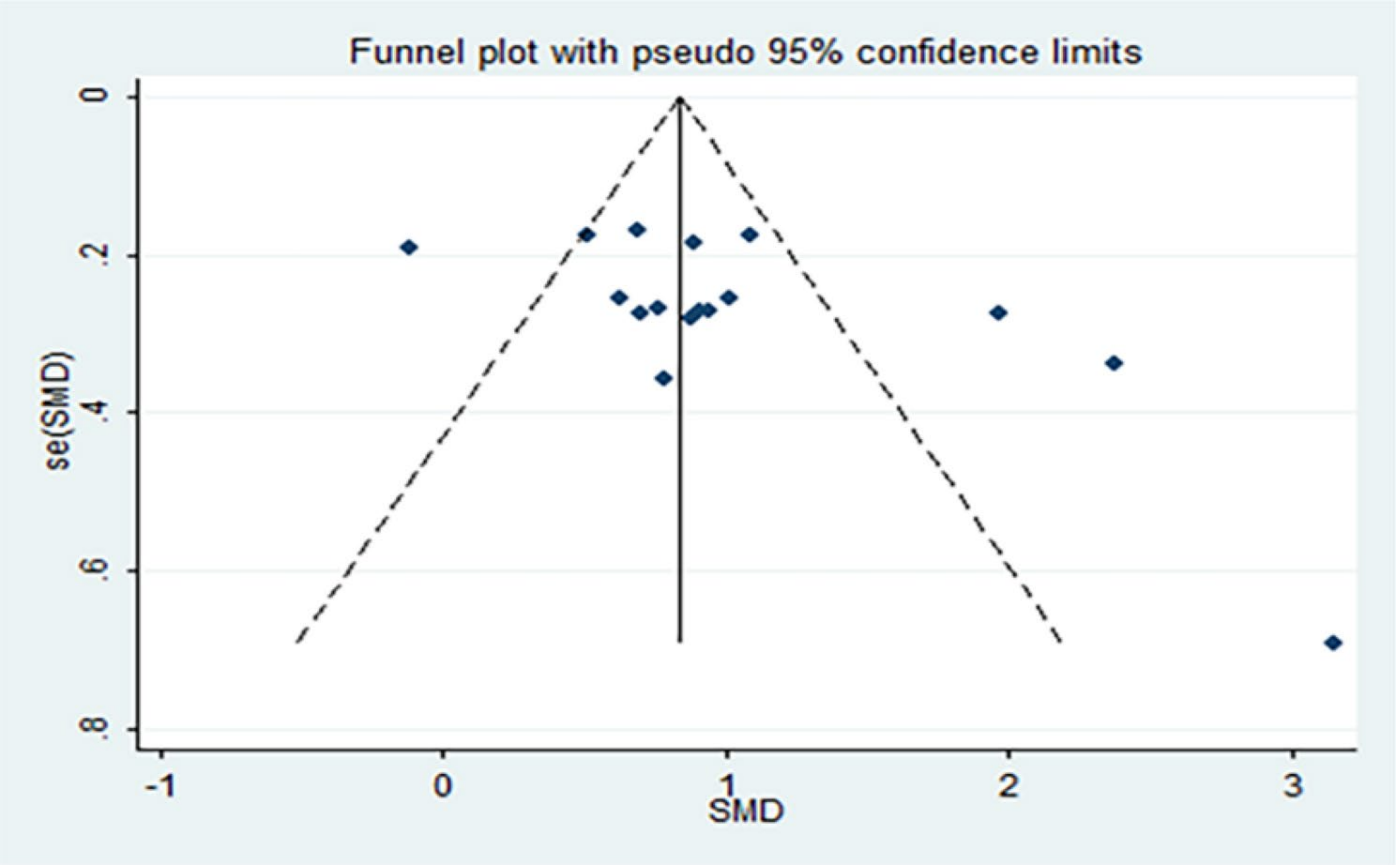
Publication bias test funnel plot

We performed a sensitivity analysis using a switching effects model and by individually removing studies. The change in effect size was not significant according to the switching effects model. However, removing the studies by Qiao and Pei et al. [16, 20]. led to a more noticeable change in effect size. We created a funnel plot for the included literature to test for publication bias, as shown in Fig. 8, and conducted Egger’s test. The results, t = 1.66, *P* = 0.024, suggest the potential for publication bias. To further assess the stability of the results, we applied the trim-and-fill method and performed seven hypothetical studies. The combined results remained statistically significant, indicating stable aggregate outcomes.

## Discussion

This study included 16 studies. Due to ethical concerns about the intervention experiments, all included studies did not blind participants, leading us to assess them as high risk in the bias risk assessment. However, we noted that in other meta-analyses, if the included studies indicated that the patient had signed an informed consent form, it could be rated as low risk in the assessment of whether the participant was blinded. Three studies implemented blinding for outcome assessors. Conversely, not blinding outcome assessors could lead researchers to be more inclined to report positive expected results, creating bias. All 16 studies clearly defined the inclusion and exclusion criteria, and they provided basic information about the literature, intervention content, outcome indicator assessment tools, and results.

The results of this study indicate that OEP can alleviate psychological FOF among older adults, which is consistent with the findings of Yu [11] and others. They conducted a descriptive analysis of two studies, and their results showed that the group-based OEP can reduce FOF in older adults. The group-based OEP promotes mutual encouragement among older adults in care facilities, enhancing their enthusiasm and adherence to the training. Research by Leem et al. [16] included the effects of individual OEP exercises and a combination of OEP with action observation training on FOF in older adults. Their study also compared the effects of the Otago Exercise Program (OEP) combined with action observation training versus OEP alone on fear of falling (FOF) in older adults. The results indicated that the combined training had a superior effect (-5.7 ± 2.11) compared to OEP alone (-5.0 ± 1.94). According to their findings, the difference between the effects of combined training and OEP alone was minimal. Each group in their study included only ten older adults. We believe that combined training extends the exercise duration, and it remains to be seen whether watching videos and prolonged training sessions might reduce exercise adherence in older adults. Further large-sample randomized controlled trials are needed to explore this issue. Currently, OEP alone appears to be more time-efficient and cost-effective, achieving satisfactory results compared to combined training. Future studies should explore the impact of combined versus individual training on compliance among older adults. The overall results showed considerable heterogeneity (I²=81%), which we attributed to clinical heterogeneity. In our study, 3 studies involved older adults with joint diseases, 6 studies involved older adults with hemiplegia, and 7 studies involved older adults in community or nursing institutions. The difference in research subjects might lead to varied intervention outcomes. Therefore we conducted subgroup analyses depending on the intervention population. The results showed that OEP had the greatest effect on older adults in the community and in institutions, followed by hemiplegic patients, but the difference in effects between the two groups was minimal, with an SMD difference of only 0.01, which we considered negligible. The intervention effects in older adults with osteoarthritis are less favorable compared to the other two groups of older adults. The reason for this difference is that in this group of older adults, they are not bothered by diseases and training in a group can increase motivation and compliance with the training. Hemiplegic patients have a strong desire for rehabilitation, hoping to recover as much as possible to their preillness state, leading to high motivation and adherence to exercise. In contrast, older adults with osteoarthritis may be reluctant to train due to pain caused by their condition. The OEP includes resistance exercises, which can cause pain during these activities, resulting in improper training and suboptimal outcomes. Ángeles et al. conducted a quasi-experimental study on 498 community-dwelling individuals aged 65–80 years in accordance with the OEP [30]. Their results indicated that FOF is a risk factor for falls in older adults, and that OEP can reduce FOF. Differences in outcome measurement tools might also contribute to heterogeneity, suggesting that future studies should use standardized measurement tools. Methodological heterogeneity, differences in intervention duration, and differences in intervention frequency among studies might also lead to varied outcomes. All included studies had a single intervention duration of more than 30 min, corroborated by Chiu et al. [31], whose results demonstrated that training sessions longer than 30 min are most effective for improving balance in older adults, as recommended in the OEP manual. Additionally, we conducted subgroup analyses based on the intervention period and frequency. The results indicate that interventions lasting longer than 16 weeks, with more than two sessions per week yielded better outcomes, aligning with the OEP manual’s recommendation of at least three sessions per week for 24 weeks. Our study included six interventions lasting more than 16 weeks, two for 16 weeks, and four for 24 weeks. Subgroup analysis revealed that 24-week outcomes surpassed 16-week outcomes; hence, we believe that training should last for at least 24 weeks, as suggested by the OEP manual. The OEP is a progressive training regimen, with a direct relationship between the intervention duration and its effectiveness. Generally, a longer intervention period allows for extended phases at each stage, enabling older adults to adapt well to the current training intensity before moving to the next phase. Shorter intervention periods often result in the study ending before the expected training intensity is reached, or older adults may be subjected to higher intensity training before they have adequately adapted. In the publication bias tests, Begg’s test showed no publication bias, but Egger’s test indicated some bias, suggesting that our results might be unstable due to publication bias. Thus, we used the trim-and-fill method to further verify the stability. The effect size remained significant and greater than zero after including seven hypothetical studies in the trim-and-fill analysis, with no reversal of the results, indicating the stability of our findings.

Our study also has limitations. The included studies did not blind participants, potentially affecting the quality and outcomes of the research and leading to results that might not align with reality. Additionally, due to the subjective nature of outcome measures derived from questionnaires and the lack of blinding among intervention subjects, measurement bias could lead to skewed results. Another limitation is the use of different measurement tools, a potential source of heterogeneity. Sensitivity analyses excluding studies by Qiao and Pei showed significant changes in effect size, identifying a source of heterogeneity. Furthermore, since follow-up durations varied, this study did not explore the long-term effects of OEP on older adults, which future research could investigate.

## Conclusion

Current evidence suggests that OEP can alleviate psychological FOF in older adults, with interventions lasting more than 24 weeks, occurring more than twice a week, and has demonstrated high effectiveness when applied among older adults in community settings or elder care institutions. Currently, a variety of assessment tools are used in studies; future research is recommended to employ uniform measurement tools. The psychological issues related to falling in older adults are as important as the falls themselves. Future efforts should focus more on the psychological aspects of falls among older adults, and more high-quality, large-sample studies should be conducted to provide stronger evidence-based support.

## Data Availability

Data from this study are provided in the manuscript and can also be obtained by contacting the authors.

## References

[1] Legters K (2002). Fear of falling. Phys Ther.

[2] Xiong W, Wang D, Ren W, Liu X, Wen R, Luo Y (2024). The global prevalence of and risk factors for fear of falling among older adults: a systematic review and meta-analysis. BMC Geriatr.

[3] Friedman SM, Munoz B, West SK, Rubin GS, Fried LP (2002). Falls and fear of falling: which comes first? A longitudinal prediction model suggests strategies for primary and secondary prevention. J Am Geriatr Soc.

[4] Ho LYW, Lai CYY, Lai CKY, Ng SSM (2024). Fatigue predicts level of community integration in people with stroke. Top Stroke Rehabil.

[5] García-Gollarte F, Mora-Concepción A, Pinazo-Hernandis S (2023). Effectiveness of a supervised Group-based Otago Exercise Program on Functional Performance in Frail Institutionalized older adults: a Multicenter Randomized Controlled Trial. J Geriatr Phys Ther.

[6] Campbell AJ, Robertson MC, Gardner MM, Norton RN, Tilyard MW, Buchner DM (1997). Randomised controlled trial of a general practice programme of home based exercise to prevent falls in elderly women. BMJ.

[7] Montero-Odasso M, van der Velde N, Martin FC et al. World guidelines for falls prevention and management for older adults: a global initiative [published correction appears in Age Ageing. 2023;52(9):] [published correction appears in Age Ageing. 2023;52(10):]. Age Ageing. 2022;51(9):afac205. 10.1093/ageing/afac205. 10.1093/ageing/afac205 PMC9523684 36178003

[8] Mangione KK, Darreff H, Welsh M (2023). Feasibility of a modified Otago Exercise Program for older adults with cognitive vulnerability. J Appl Gerontol.

[9] Albornos-Muñoz L, Moreno-Casbas MT, Sánchez-Pablo C (2018). Efficacy of the Otago Exercise Programme to reduce falls in community-dwelling adults aged 65–80 years old when delivered as group or individual training. J Adv Nurs.

[10] Wu S, Guo Y, Cao Z, et al. Effects of Otago exercise program on physical function in older adults: a systematic review and meta-analysis of randomized controlled trials. Arch Gerontol Geriatr Published Online May. 2024;3. 10.1016/j.archger.2024.105470. 10.1016/j.archger.2024.105470 38718487

[11] Peng Y, Yi J, Zhang Y, Sha L, Jin S, Liu Y (2023). The effectiveness of a group-based Otago exercise program on physical function, frailty and health status in older nursing home residents: a systematic review and meta-analysis. Geriatr Nurs.

[12] Yi M, Zhang W, Zhang X, Zhou J, Wang Z (2023). The effectiveness of Otago exercise program in older adults with frailty or pre-frailty: a systematic review and meta-analysis. Arch Gerontol Geriatr.

[13] Higgins JP, Altman DG, Gøtzsche PC, et al. The Cochrane collaboration’s tool for assessing risk of bias in randomised trials. BMJ. 2011;343:d5928. 10.1136/bmj.d5928. Published 2011 Oct 18. 10.1136/bmj.d5928 PMC3196245 22008217

[14] Arkkukangas M, Söderlund A, Eriksson S, Johansson AC (2019). Fall preventive Exercise with or without Behavior Change Support for Community-Dwelling older adults: a Randomized Controlled Trial with Short-Term follow-up. J Geriatr Phys Ther.

[15] Genç FZ, Bilgili N (2023). The effect of Otago exercises on fear of falling, balance, empowerment and functional mobility in the older people: Randomized controlled trial. Int J Nurs Pract.

[16] Leem SH, Kim JH, Lee BH (2019). Effects of Otago exercise combined with action observation training on balance and gait in the old people. J Exerc Rehabil.

[17] Lytras D, Sykaras E, Iakovidis P, Komisopoulos C, Chasapis G, Mouratidou C (2022). Effects of a modified Otago exercise program delivered through outpatient physical therapy to community-dwelling older adult fallers in Greece during the COVID-19 pandemic: a controlled, randomized, multicenter trial. Eur Geriatr Med.

[18] Mat S, Ng CT, Tan PJ (2018). Effect of Modified Otago exercises on postural balance, fear of falling, and fall risk in older fallers with knee osteoarthritis and impaired gait and balance: a secondary analysis. PM R.

[19] Zou Z, Chen Z, Ni Z, Hou Y, Zhang Q (2022). The effect of group-based Otago exercise program on fear of falling and physical function among older adults living in nursing homes: a pilot trial. Geriatr Nurs.

[20] Qiao YH, Cao HJ, Lian Y, Ni YC (2017). Follow-up study on Otago exercise program on fear of falling in central hemiplegia patients in communities. West China Med J.

[21] Liu H, Ji DH, Chi X, Gu LM, Bai CJ, Zhao QY (2019). Effect of Otago Exercise Programme on balance ability and fear of falling in elderly patients with knee arthroplast. Chin Nurs Manage.

[22] Zhou Q, Yan DY (2021). The effect of Otago exercise program on elderly patients with fear of falling. Chin Clin Nurs.

[23] Tang LJ, Yue LC, Liu CX, Wu L, Peng YY (2016). The effect of Otago exercise on fall fear and balance ability of discharged patients with Parkinson’s disease. Chin J Rehabilitation Med.

[24] Zhang XB, Wang XL, Yang XZ, Meng YC, Huo LZ, Zhang HX, Liu L, Wang LL (2023). Effect of Otago Exercise based on empowerment theory in Elderly Stroke patients with fear of falling. Med Innov China.

[25] Li Y, Cheng Y, Zhao LR, Hu YQ (2024). Effect of Otago Exercise Programme on fear of falling in elderly patients with stroke. Chin J Nurs.

[26] Pei XY, Lu MQ, Wang FL, Zhang XL, Zhang P, Xing FM (2023). Effect of Otago exercise training on fear of falling in older adults. Chin Nyrsing Res.

[27] Zhao QY, Ji DH, Zhang Y (2019). Effect of Otago Exercise Programme on fear of falling in Elderly patients with femoral Neck fractures. J Changchun Univ Chin Med.

[28] Qiu HZ (2016). Observation on the preventive effect of Otago exercise on the fear of falling in stroke patients. J Chin Physician.

[29] Gu Y, Shen Y, Yu XP, Zhu Y (2020). Application effect of Otago exercise programme in the elderly with the fear of falling. Chin Nurs Res.

[30] Ángeles CM, Laura AM, Consuelo CM (2022). The effect that the Otago Exercise Programme had on fear of falling in community dwellers aged 65–80 and associated factors. Arch Gerontol Geriatr.

[31] Chiu HL, Yeh TT, Lo YT, Liang PJ, Lee SC (2021). The effects of the Otago Exercise Programme on actual and perceived balance in older adults: a meta-analysis. PLoS ONE.

